# Patient perspectives on engagement in decision-making in early management of non-ST elevation acute coronary syndrome: a qualitative study

**DOI:** 10.1186/s12911-017-0555-9

**Published:** 2017-11-28

**Authors:** Todd Wilson, Jean Miller, Sylvia Teare, Colin Penman, Winnie Pearson, Nancy J. Marlett, Svetlana Shklarov, P. Diane Galbraith, Danielle A. Southern, Merril L. Knudtson, Colleen M. Norris, Matthew T. James, Stephen B. Wilton

**Affiliations:** 1 Department of Community Health Sciences, Cumming School of Medicine, University of Calgary, 3280 Hospital Drive NW, Calgary, AB T2N 4Z6 Canada; 2 Patient and Community Engagement Research program, O’Brien Institute of Public Health, Cumming School of Medicine, 3280 Hospital Drive NW, Calgary, AB T2N 4Z6 Canada; 3 Department of Cardiac Sciences, Cumming School of Medicine, University of Calgary, 3280 Hospital Drive NW, Calgary, AB T2N 4Z6 Canada; 4 Libin Cardiovascular Institute of Alberta, Cumming School of Medicine, University of Calgary, GE64 3280 Hospital Drive NW, Calgary, AB T2N 4Z6 Canada; 5 Faculty of Nursing, University of Alberta, 4-171 Edmonton Clinic Health Academy, TCG 1C9, Edmonton, AB Canada

**Keywords:** Acute coronary syndrome, Shared decision making, Grounded theory, Decision aid, Patient engagement

## Abstract

**Background:**

Surveys of patients suggest many want to be actively involved in treatment decisions for acute coronary syndromes. However, patient experiences of their engagement and participation in early phase decision-making have not been well described.

**Methods:**

We performed a patient led qualitative study to explore patient experiences with decision-making processes when admitted to hospital with non-ST elevation acute coronary syndrome. Trained patient-researchers conducted the study via a three-phase approach using focus groups and semi-structured interviews and employing grounded theory methodology.

**Results:**

Twenty patients discharged within one year of a non-ST elevation acute coronary syndrome participated in the study. Several common themes emerged. First, patients characterized the admission and early treatment of ACS as a rapidly unfolding process where they had little control. Participants felt they played a passive role in early phase decision-making. Furthermore, participants described feeling reduced capacity for decision-making owing to fear and mental stress from acute illness, and therefore most but not all participants were relieved that expert clinicians made decisions for them. Finally, once past the emergent phase of care, participants wanted to retake a more active role in their treatment and follow-up plans.

**Conclusions:**

Patients admitted with ACS often do not take an active role in initial clinical decisions, and are satisfied to allow the medical team to direct early phase care. These results provide important insight relevant to designing patient-centered interventions in ACS and other urgent care situations.

## Background

Acute coronary syndrome (ACS) is a major cause of mortality and morbidity in Canada, with in-hospital mortality in up to 5% of patients [1]. Non-ST elevation ACS can be managed using either an invasive approach (involving use of coronary angiography and percutaneous coronary intervention and/or cardiac surgery for revascularization) or a conservative approach (employing medical therapies and reserving invasive procedures only for people with signs of ongoing cardiac ischemia despite medical management). Randomized trials show early invasive management reduces the risk of recurrent myocardial infarction, re-hospitalization, and improves long-term survival in appropriately selected high-risk individuals compared with conservative management for ACS [2–6]. Accordingly, current guidelines recommend early invasive management for high-risk individuals with ACS [7]. However, for those patients not considered to be high-risk, the optimal time to proceed to invasive management and re-vascularization or persist with medical therapy is uncertain [6, 8]. This decision point has implications for patient outcomes such as mortality, bleeding and kidney injury events, as well as for health care system costs.

The patient’s role in treatment decisions in the setting of ACS has recently begun to receive more attention. Krumholz et al. conducted a survey of 6636 patients from the United states 24 to 72 h after admission for an acute myocardial infarction (MI) and found a majority of patients wanted an active role in their treatment decisions [9]. Another study found that while most patients did not want to make treatment decisions themselves, the vast majority wanted their preferences to be taken into account by their physician [10]. However, the patient’s capacity to make decisions in the emergency setting may preclude them from actively participating in treatment decisions and, in such cases, patients have been found to be more willing to defer decisions entirely to their physicians [11].

Patient targeted decision aids are widely used in outpatient and ambulatory settings to support patients in medical decision-making [12]. However, little research has been conducted into patient perspectives on their engagement in the early decision-making processes for ACS. This qualitative study was designed to explore the experiences of patients on their participation in the decision making process for early non-ST elevation ACS care, and further explored patient perspectives on potential use of decision aids when admitted to hospital with ACS.

## Methods

We used methods previously reported by the Patient and Community Engagement Research (PaCER) program [13]. The PaCER methodology is based on grounded theory, whereby qualitative data is collected iteratively, analyzed for themes, and used to plan subsequent phases of the research process through the use of trained patient researchers. This theoretical approach relies on the underlying concept of generating new theory as data are collected, as opposed to collecting data to test an existing theory [13–15].

### Patient-researchers –patient and community engagement researchers (PaCERs)

Data collection and analysis were performed by four trained PaCERs. The two lead PaCERs (JM, ST) had a history of chronic disease and completed the formal PaCER internship in patient engagement research methods and the grounded theory framework, and had conducted previous projects using these methods [13]. They were supported by an additional two PaCERs with personal experiences with heart disease and training in patient engagement (WP, CP). Oversight at each phase was provided by PaCER and APPROACH faculty.

### Participant recruitment

Potential study participants were identified from the Alberta Provincial Project for Outcome Assessment of Coronary Heart Disease (APPROACH) registry [16]. We used purposive sampling to ensure the study represented a broad range of ACS patient ages and was approximately balanced by sex. Inclusion criteria were hospitalization with non-ST elevation ACS within the last year, fluency in English, residency in the local area, and having previously provided informed consent to be contacted for research purposes.

### Data collection

Data collection followed an iterative, three phase (Set, Collect, Reflect) approach that has been previously published [13, 17, 18].

The Set, co-design phase consisted of a single focus group of ACS patients. Participants were asked to share their positive and negative experiences with their last hospitalization for ACS and how the treatment they received unfolded to explore the scope of responses, language used and choice and modification of data collection. In addition, participants were asked what could have made for a better experience. Discussion points were documented on flip chart pages and the session was recorded.

Based on findings during the Set, co-design phase, the Collect phase consisted of a focus group followed by individual interviews. Participants in the focus group were asked to speak about the treatment decision-making process and their awareness of, and interest in being involved in treatment options. In addition, the participants explored the idea of being provided with a decision aid tool to help involve them in the decision-making process.

The interview guide to be used for individual interviews was informed by the analysis of audiotape and focus group notes. Interviews were conducted in pairs with one lead patient researcher accompanied by one of two patient researchers with a history of heart disease. Individual interviews were conducted with ACS patients not involved with the Set phase focus groups.

Data about the clinical presentation, risk factor profile, and in-hospital management were extracted from the APPROACH database. In APPROACH, the classification of ACS represents the attending physician’s final discharge diagnosis.

### Data analysis

Audiotapes and interviewer’s notes from each interview were used to create a descriptive document, which was analyzed independently by two PaCERs. Each stage of data collection was coded to explore points that spoke to decision-making and value of a decision aid. The researchers compared and contrasted their points and through a collaborative process five categories were identified: 1) awareness of treatment decisions; 2) interest in being more involved in treatment decisions; 3) information they were given; 4) interest in more information about treatment options; and 5) perceived value of a decision aid tool.

The five categories identified in the Collect phase were taken to the Reflect focus group, which consisted of participants from either the Set or Collect phase. During this phase, participants collaborated around the data analysis and reflected on the fit between what they had said and what was reported. In addition, some information was explored in more detail and recommendations were developed. The focus group ended with a reflection on what they were taking away from their involvement in the study.

No further focus groups or interviews were planned once the research team was confident saturation had been reached and no new themes were emerging from further patient interviews and focus groups.

The funding source did not have a role in this study.

## Results

Of 75 eligible patients identified from the APPROACH database and approached, 20 agreed to participate in the study. Their characteristics are described in Table 1. The median age of participants was 68.5 years, 60% were men, 70% were hospitalized with a non-ST elevation myocardial infarction, all received cardiac catheterization and 55% went on to receive percutaneous or surgical revascularization.

**Table 1:** Patient Characteristics

Characteristic	No. of Patients (*n* = 20)
Age:	
Age at ACS presentation (median, range)	68.5 yrs. (51.3 yrs. to 87.5 yrs)
< 60	3
60–74	11
75 and older	6
Sex:	
Female	8
Male	12
Length of Hospital Stay (median, range)^a^	4 days (2 days to 78 days)
Admission Diagnosis	
ACS **–** NSTEMI	14
ACS - Unstable angina	4
Other^b^	2
Cardiac risk profile and history	
Hypertension	10 (50%)
Diabetes mellitus	3 (15%)
Smoking (Current / Past)	5 (25%) / 5 (25%)
Dyslipidemia	13 (65%)
Family history of premature coronary disease	9 (45%)
Previous coronary disease diagnosis	13 (65%)
Previous ACS	7 (35%)
Previous percutaneous coronary intervention	6 (30%)
Previous coronary artery bypass surgery	1 (5%)
Congestive heart failure	2 (10%)
ACS Management from Last Admission	
Cardiac catheterization	20 (100%)
Percutaneous coronary intervention	7 (35%)
Coronary artery bypass surgery	4 (20%)

^a^ Length of hospital stay not reported in seven patients

^b^ 2 participants had a reason for their index hospitalization that was not ACS (1 stable angina, and 1 sudden cardiac arrest), but had suffered a previous ACS

*ACS* acute coronary syndrome, *NSTEMI* non-ST elevation myocardial infarction

The following themes regarding experiences from their hospital admission and the decision-making process for ACS treatment were identified from participants.

### Perception of the emergency hospital admission

Participant stories of their hospitalization experience with ACS began with the onset of their symptoms and subsequent arrival at the emergency department and admission to hospital. They found themselves in an unexpected and potentially life-threatening situation and in some cases an outright crisis. They were shocked, scared, and felt out of control. Participants also recounted how quickly events unfolded once they arrived at the hospital (Table 2).

**Table 2:** Perception of the emergency hospital admission

Shocked, scared and out of control	
“This was the most significant emotional event in my life” …“a wake-up call”. “It hit me square in the head”…“not being invincible was the biggest shock” (Participant 15)	
“The guy upstairs flips the switch” …. “What am I doing here?” (Participant 14)	
“I’m from a family of *events* and this was definitely not an event: it was an *attack”*. “I felt like a deflated balloon when it happened” (Participant 18)	
Rapidly unfolding interventions	
“I guess I don’t know why it was decided to admit me”….“I zipped away upstairs to the heart unit” (Participant 19)	
“Things went really fast” … “(they) hooked me up to everything” (Participant 17)	
“(I was) treated as number one priority” (Participant 12)	

### Patients’ views on involvement in ACS treatment decision-making on emergency hospital admission

Participants felt they had not been involved in making treatment decisions when admitted to hospital and they were often unaware of different treatment options. Patients acknowledged that as specialists in their field, cardiologists were in the best position to make treatment decisions. Participants described that once cardiologists had considered the options and selected the treatment that was best for them, the recommendation would be presented to them and they were given the opportunity to consent to the recommended treatment. On reflection they thought this approach was understandable given the life-threatening nature of their illness. However, patients felt treatment decisions were made *for* them rather than *with* them (Table 3).

**Table 3:** Treatments made *for* them*,* rather than *with* them

“They’ve looked at the options and have decided what is best for you and then they tell you that and you decide if you will do it or not” (Participant 8)
“I don’t need a bunch of options…. I do what the expert believes is the solution for me”…“If I cut my finger, fix it…don’t give me options”, for example a tourniquet or amputation, go with the focused solution (Participant 15)
“My thinking is I’m having trouble, these are professionals – I let them do what they do – I put my life in their hands. When I worked I was the expert in my field called in due to a problem, took necessary action – did my thing didn’t rely on client” (Participant 16)
“There would be a lot going on behind the scene: doctors talking to doctors etc. as they make the decision they think is best” (Participant 11)
(The doctors got together) “They voted among themselves if I would survive the surgery or not” (Participant 17)

### Feeling incapable of participating in decision-making

Study participants thought that given the stressful life-threatening situation in which they found themselves, they would not have been capable of participating in complex decision-making. In this situation they relied on someone else to make the decisions. Realizing that decisions had to be made quickly, they put their trust in the cardiologists (Table 4).

**Table 4:** Feeling incapable of participating in decision-making

“You have to realize that after you have a heart attack you barely know your name, you have no memory, you’re scared, everything up there is scrambled – you’re in total disbelief (that you’ve had a heart attack)”… “there are very few decisions that you can make that would be the right ones” (Participant 18).
“They took the decision away from me, and I’m glad as that reduced my stress” (Participant 11)
“You don’t have a lot of time – your body is not giving you lot of time for decisions before things go wrong so you trust them to make the right decisions” (Participant 18).

### Relinquishing control to experts in a presumed emergency

Participant’s were comfortable with their cardiologists making treatment decisions and were willing to turn their decisions over to the experts. In fact, many participants felt this should be the normal course of action. Patients relied on the cardiologists’ expertise and experience, putting their trust in them (Table 5).

**Table 5:** Turning their bodies over to the experts

“I accepted it (the doctor’s decision) because I was in shock. I just went along with it which is not what I usually do…I’m Irish. Something made me trust them.” (Participant 13)
“I assumed they did what they needed to do” (Participant 16)
“I just wanted the problem fixed”…“I would rely on my doctor’s advice more than what I think is appropriate” (This participant trusted the doctors to know best and this trust stemmed from their confidence.) (Participant 14).
“I expected the surgeon knew what he was doing and did exactly what he said he would do…No question there” (Participant 15)
“They are in a better position to choose options.”….. “This isn’t necessarily bad as they have better knowledge and experience with others in the same situation.” (Participant 8)

### Potential role of a patient-targeted decision aid to support ACS treatment decision-making

Patients were asked to reflect on the potential role of a decision aid to support their engagement in decision-making when admitted to hospital with ACS. The predominant view was that given the life-threatening situation and their mental and emotional state, such a tool would not have been helpful in the initial phase of their admission. However, some participants voiced a different perspective that this may be helpful, particularly once past the initial acute phase of care, or when involving other care providers or family members in treatment decisions. These patients felt an individualized shared decision-making tool would begin to address their specific information needs and would promote collaborative decision-making. The tool would also help them absorb all they needed to know before they went home and guide them on what specific questions to ask in their follow-up medical appointments (Table 6).

**Table 6:** Patients’ views on a decision aid for early ACS treatment decision-making

On admission to emergency care	
One participant said that early on they felt very foggy and was in no position to make decisions…and even after emerging from the fog the participant didn’t think more information would have been helpful (Participant 14)	
“Yes, they can, like a sketching form, this is the % that this will work or not work”. When asked whether their head was too foggy to make decisions and the doctors should do it, this participant said: “That’s one way of doing, but I had the back-up: one daughter is a chiropractor the other has brains too”. (Participant 17)	
“In emerg (sic) it’s possible you’re not understanding things all that much but it would have been helpful somewhere along the line”. (Participant 19)	
Once past the life-threatening stage	
It would “tell you this is what happened, this is what we did, and what we found, and what medications we are giving you and this is what they are for”. (Participant 19)	
“Information isn’t volunteered….you have to know what to ask” (Reflect focus group participant).	
One interviewee indicated they would like to reduce some of the medications they were taking but didn’t know how to go about this. (Participant 19)	

## Discussion

In this qualitative study, participants who had been recently hospitalized with an ACS described the emotional shock and loss of control that accompanied their event, yet reported generally positive experiences with their care. Importantly, with respect to their role in decision-making, the majority of participants were unaware of the decision to pursue an early invasive versus medical management approach to treatment of their ACS, and did not describe being involved in this decision. Participants acknowledged that decisions were largely made for them rather than with them during their care, yet felt this was appropriate given the circumstances. Most participants were grateful that important decisions had been made for them, as they felt they would not have been able to make sound decisions in the early acute phase of their admission. Some participants expressed a need for health professionals to better understand their individual circumstances; provide more explanation about what lay ahead, and clearer explanations about their treatment, particularly later in their admission. These findings identify important themes that are relevant when considering strategies to engage patients in decision making for ACS care.

Little research has been conducted into the involvement of patients in the decision-making process during the initial admission period of an acute illness. A recent qualitative study found that patients faced with decision-making in the emergency department felt unbearable pressure and a compromised ability to absorb new information. In these situations patients were able to relieve this pressure by placing their trust in the healthcare provider and transferring the decision to them. [11] For the most part, the patients in our study were relieved that specialized professionals stepped in and made the decisions in the acute setting. Similar to other research, the findings of our study are likely explained by the life-threating nature of ACS events that leave patients mentally and emotionally unable to participate in complex decision-making.

The study’s findings also suggest that the extent to which patients want to be involved in decision-making is likely to vary, with some wanting more involvement than others. Wang et al. found that the extent to which patients want to be involved in decision-making is related to previous experience or a priori knowledge of their health condition [11], in addition to their level of acuity. During the emergency stages of ACS admission, most patients felt there would be limited benefit from patient-targeted decision aids. This suggests that ACS decision support tools, for example to help tailor the management approach to patient risk, are likely best targeted to clinicians rather than patients. Other studies have suggested shared decision-making can be an effective strategy in emergency care. A systematic review of fives studies on engaging patients in shared decision-making in the emergency setting reported positive results for the use of decision support interventions targeted at patients and patient surrogates, improvements in patient or surrogate engagement in decision-making, patient knowledge and satisfaction of care. This review suggested a positive impact of eliciting patient preferences and values towards treatment options in emergency care, and suggests a potential role for patient involvement in decision-making may remain in ACS care (17). This may be most valuable once patients are past the crisis situation, which is when participants in our study showed the most interest for greater involvement in decision-making. In addition, this is where they expressed the greatest interest in tools that could address their personal values related to the potential harms and benefits of treatment choices, including their medications. Such tools could be tailored to their individual circumstances and would support their learning in the non-emergency hospitalization phase and beyond.

Participants in our study were interested in more information on their medication management before being discharged from hospital, including how each medication will benefit them. Improving delivery of this information to patients may help engage them in secondary prevention of cardiovascular disease. This is similar to findings from qualitative research in patients’ perspectives of care for osteoarthritis, where patients expressed a need for specific self-management strategies that align with the stages of OA severity, along with specific information about when they should seek further help [17]. Together these studies suggest the information patients typically receive may not be at the level of specificity they require.

Limitations of this study include the small sample size common to qualitative studies of this nature. The sample is within the expected range for saturation in grounded theory studies. While we continued to recruit patients until thematic saturation was reached, the generalizability of the results to other ACS patients and care settings is unknown. Importantly, all patients received an invasive management approach for ACS and were at high risk for adverse outcomes based on their admission characteristics. This may have made physicians more confident in treatment recommendations and reduced the need to engage patients in treatment decisions. In addition, our sampling strategy did not select patients based on clinical outcomes after discharge. Though participants had a variety of demographic and clinical characteristics, and differed in their initial presentation and management, additional themes may have emerged if our sample included patients having experienced more adverse outcomes. It is possible that a critical case analysis would have revealed that such patients had different perceptions of decision-making in the initial phase of their ACS care than those with positive outcomes. We lacked follow-up clinical data to perform such an analysis. Our findings may not be generalizable to patients treated conservatively or in scenarios where there is less certainty about the benefits versus risk of an invasive versus conservative management approach. In addition, recruitment for this study was focused on patients themselves and did not include family members who are often key advocates when patients are unable to participate in decision-making.

## Conclusion

In conclusion, we used a grounded theory approach to engage patients to share their views on their hospital admission, subsequent decision-making process, and the potential role for a decision aid in ACS care. We found that most patients did not feel they had taken an active role in initial care decisions, instead allowing the medical team to direct early phase care. Importantly, in general our patients did not feel capable of participating actively in decision-making early in their presentation, and were confident in decisions taken by the medical team during the emergency phase of care. Although patients felt that a patient-targeted decision aid may not be useful during the initial period of emergency admission, they felt such tools may be beneficial once they have stabilized, have the ability to participate in treatment decisions and can use the tool to tailor their treatment choices in the non-emergency hospitalization phase and subsequent discharge.

## Abbreviations

ACS: Acute Coronary Syndrome
APPROACH: Alberta Provincial Project for Outcome Assessment of Coronary Heart Disease
PaCER: Patient and Community Engagement Research program
PaCERs: Patient and Community Engagement Researchers
